# Thermodynamic Basis of Paton’s Rate Theory of Drug Action: Occupation Theory as Its Steady-State Limit

**DOI:** 10.7759/cureus.117367

**Published:** 2026-10-02

**Authors:** Robert B Raffa

**Affiliations:** 1 School of Pharmacy, Temple University, Philadelphia, USA

**Keywords:** drug-receptor interaction, occupation theory, paton, rate theory, thermodynamics, transition state

## Abstract

Rate theory does not contradict occupation theory; instead, it contains it as a special case. The ‘rate theory’ of drug action proposes that drug-induced pharmacologic stimulus (and consequent effect) tracks better with the rate of drug-receptor complex formation than with the fraction of receptors occupied at equilibrium, as proposed in classical ‘occupation theory.’ But rate theory was initially framed phenomenologically, in terms of two empirical rate constants (k_1_, k_2_) rather than from an underlying physical basis. Here, we show that rate theory’s main postulate follows directly from standard chemical transition-state theory once k_1_ and k_2_ are recognized as transition-state rate constants governed by the free energies of association, ΔG^‡^_1_, and dissociation, ΔG^‡^_2_. This reframing reveals the classical equilibrium affinity constant, K_A_, to be the difference between the two activation barriers, ΔG^‡^_1_ - ΔG^‡^_2_; shows that a drug's pharmacologic characterization as a full agonist, partial agonist, or antagonist is determined by the reverse barrier ΔG^‡^_2_ (equivalently, by 1/residence time) rather than by affinity; shows that occupation theory is the steady-state limit of rate theory; and can be easily extended to encompass access-limited pharmacokinetics and to the separation of ligand binding from transduction. The latter provides a thermodynamic account of why affinity and efficacy are not in general independent quantities. The derivation is extended to demonstrate how it unifies rate kinetic theory with modern approaches to drug-receptor residence time in drug discovery. These concepts are illustrated, including a free-energy reaction-coordinate diagram, a free-energy well with residence time, and a simulated time-course comparison of stimulus versus occupancy.

## Introduction

The central goal of this study is to demonstrate that Paton's rate theory [1-4] is not a rival empirical alternative to occupation theory [5-13], but instead is a special case of a more general thermodynamic [14] and transition-state description of drug-receptor interactions in which occupation theory is the steady-state limit. It is only a generalizable analytical framework, since actual receptor systems can involve multiple conformational states and parallel pathways.

For the derivations, the starting point was Paton's 1961 [2] elementary bimolecular kinetic schema of the interaction between drug molecule A and receptor R leading to drug-receptor complex AR according to the canonical representation $\mathrm{A} + \mathrm{R} \rightleftharpoons \mathrm{AR}$, involving two rate constants k_1_ and k_2_. Each rate constant is re-expressed here in Eyring’s [15] transition-state form, $k = \kappa (k_{\mathrm{B}}T/h) \cdot \exp(-\Delta G^{\ddagger}/RT)$, and combined with Paton's definitions of stimulus ($S = \mathrm{const} \times k_1[\mathrm{A}][\mathrm{R}]$) and reciprocal potency ($k_{\mathrm{e}} = k_2/k_1$) to obtain the free-energy relationships. The recovery of occupation theory as a steady-state limit follows by setting $\mathrm{d}[\mathrm{AR}]/\mathrm{d}t = 0$ in the same kinetic schema and comparing the resulting flux expression with the standard occupancy expression. The two extensions were obtained by inserting an additional activated step in series (access-limited kinetics) [3,16], or in sequence (binding-then-transduction) [17], and re-deriving the corresponding free-energy relationships by the same thermodynamic-cycle argument. Although this presentation provides a theoretical derivation rather than an empirical study, the results have the following three practical implications for contemporary pharmacology and drug discovery.

First, the results provide a first-principles explanation for the now-common practice of optimizing drug-receptor residence time ($\tau = 1/k_2$) along with, or instead of, equilibrium affinity (K_A_) [18-20]. Because K_A_ is the difference between two activation barriers (ΔG^‡^_1_ - ΔG^‡^_2_), two compounds with identical affinity can have different k_1_ and k_2_, and therefore different in vivo durations, kinetic selectivity profiles [21,22], and even different qualitative pharmacological character in Paton's original sense (full agonist vs partial agonist vs antagonist). An optimization strategy that tracks K_A_ alone is, in this framework, blind to the parameter that governs much of a drug's behavior in a real, non-equilibrium tissue environment (ΔG^‡^_2_, or equivalently τ).

Second, the transition-state (Eyring-plot) approach offers a concrete, testable extension of structure-activity relationships (SARs): resolving k_1_ and k_2_ into enthalpic and entropic components [14] should, in principle, let medicinal chemists distinguish compounds that achieve a given residence time through a favorable, enthalpy-driven, specific interaction from those that achieve it through an entropy-driven one [23], a distinction that is not possible with K_A_ or τ alone.

Third, the binding/transduction extension provides a thermodynamic foundation for biased agonism [24,25]: if a single receptor can adopt more than one active conformation, each preferentially coupled to a different effector, then a ligand's efficacy toward each pathway is governed by a pathway-specific gating free energy, ΔG^‡^_gating_, rather than by a single scalar “efficacy” value [26]. This reframes biased agonism as an expected consequence of a multi-well free-energy surface rather than as a phenomenon requiring a separate model.

As demonstrated here, viewed under overarching transition-state theory, rate theory and occupation theory are not competitors. Occupation theory is the equilibrium limit of rate theory, and the parameter that principally distinguishes an agonist from a partial agonist from an antagonist is the reciprocal of the drug-receptor residence time.

## Materials and methods

For convenience, a glossary of terms and symbols used in the development is presented below (Table 1).

**Table 1:** Glossary of terms and symbols The table lists the pharmacologic, chemical, and thermodynamic concepts used as the basis for the derivations and the symbols used to represent them.

Term	Definition
Access-limited kinetics	A situation in which the observed speed of drug action is governed by how fast the drug can physically reach the receptor (e.g., diffusion, tissue partitioning) rather than by the intrinsic chemical rate of binding.
Affinity	The mutual attraction between drug (A) with its receptor (R) that leads to AR, independent of how fast it gets there.
Agonist	A drug that activates a receptor to produce a response.
Antagonist	A drug that binds to a receptor without activating it, thereby blocking access to agonists.
Association rate constant, k_1_	A number describing how quickly drug (A) and receptor (R) combine to form the complex AR; a larger k_1_ means faster binding.
Biased agonism / functional selectivity	The phenomenon in which a single drug produces different degrees of activation of different downstream signaling pathways from the same receptor.
Boltzmann constant k_B_; Planck constant h	Fundamental physical constants that appear together as the prefactor k_BT_/h in the Eyring equation.
Del Castillo–Katz mechanism	A two-step receptor schema that separates drug binding $(\mathrm{A} + \mathrm{R} \rightleftharpoons \mathrm{AR}$) from the subsequent conformational “gating” step that produces the active receptor state $(\mathrm{AR} \rightleftharpoons \mathrm{AR}^{*}$).
Dissociation rate constant, k_2_	A number describing how quickly the complex AR dissociates into free drug and free receptor; a larger k_2_ means the complex is shorter-lived.
Efficacy	How effectively an occupied receptor actually produces a biological response, as distinct from how tightly the drug is bound.
Enthalpy of activation, ΔH^‡^	The heat-related (bond-making / bond-breaking) part of the activation energy.
Entropy of activation, ΔS^‡^	The disorder-related (conformational, solvent, orientational) part of the activation energy.
Equilibrium dissociation constant, K_A_	The ratio k_2_/k_1_; a smaller K_A_ means the drug binds more tightly (higher affinity).
Eyring equation	The formula from transition-state theory that expresses a rate constant in terms of ΔG^‡^ (or ΔH^‡^ and ΔS^‡^) and temperature.
Fade	A decline in response during continued or repeated exposure to an agonist, even while the tissue still contains receptor-bound drug.
Free energy of activation, ΔG^‡^	The energy “hill” that must be climbed to reach the transition state; it dictates how fast a reaction proceeds.
Gating / isomerization step	The conformational change a receptor undergoes after drug (or other ligand) binding, which converts an inactive bound complex into the active, signaling form.
Occupation (occupancy) theory	The classical model in which a drug's effect depends only on the equilibrium fraction of receptors it has bound, not on how fast that binding happened.
Operational model of agonism	Black and Leff's mathematical framework for extracting affinity and efficacy from a concentration–response curve without assuming a specific receptor mechanism.
Partial agonist	A drug that activates a receptor, but produces a smaller maximum response than a “full” agonist, even at saturating concentration.
Rate theory	Paton's alternative model in which a drug's effect depends on the ongoing rate at which new drug–receptor complexes are being formed, not on how many there are bound at any instant.
Receptor occupancy, p_A_	The fraction of the total receptor population that currently has drug bound to it: $p_{\mathrm{A}} = [\mathrm{AR}] / R_{\mathrm{total}}$.
Reciprocal potency constant, k_e_	Paton's symbol for k_2_/k_1_, used in his 1961 paper in place of the classical affinity constant.
Residence time, t	The average lifetime of a drug–receptor complex before it dissociates: $\tau = 1/k_2$.
Standard free energy of binding, ΔG°_bind_	The overall free-energy change for $\mathrm{A} + \mathrm{R} \rightarrow \mathrm{AR}$ at equilibrium; related to K_A_ by $\Delta G^{\circ}_{\mathrm{bind}} = -RT \ln(1/K_{\mathrm{A}})$.
Stimulus, S(t)	Paton's term for the instantaneous pharmacologic output of the receptor system; in rate theory, it is proportional to the rate of complex formation, k_1_[A][R], rather than to occupancy.
Tachyphylaxis	A rapid, repeated-dose decline in a drug's effect, often faster than can be explained by simple receptor depletion.
Ternary complex model	A model of G-protein-coupled receptor activation in which the active, high-affinity state requires a three-part complex of agonist, receptor, and a coupling protein (e.g., a G protein).
Transition state / activated complex	The fleeting, highest-energy molecular arrangement that a reaction must pass through on its way from reactants to products.
Transmission coefficient, κ	A correction factor (often simplified to 1 for simplicity) for the fraction of transition-state crossings that actually go on to form product rather than revert to reactants.
Two-state (MWC) model	A model in which a receptor (or any allosteric protein) can exist in two conformations, with ligands shifting the equilibrium between them.

The difference of rate theory from occupation theory

Classical ‘occupation’ drug-receptor theory [5,6,12,26] treats the pharmacologic stimulus (leading to drug effect) as a function of the fraction of receptors occupied at equilibrium, according to $p_{\mathrm{A}} = [\mathrm{AR}]_{\mathrm{eq}}/R_{\mathrm{total}} = [\mathrm{A}]/([\mathrm{A}] + K_{\mathrm{A}})$, where K_A_ is the equilibrium dissociation constant (ratio of reverse to forward interaction rate constants, k_2_/k_1_). Occupation theory is therefore a ‘ground-state’ thermodynamic theory: that is, it asks only how the free energy of the bound complex AR compares to that of free A + R, and not the approach.

In contrast, Paton’s 1961 [2] ‘rate’ theory replaces this framework with a kinetic one, postulating that drug-induced stimulus (leading to drug effect) is proportional not to how many receptors are occupied, but instead, to how fast new AR complexes are being formed - that is, to the rate at which drug molecules cross the energy barrier to associate with the receptor (Figure 1). Paton specified the system with two rate constants: k_1_, the association rate constant, and k_2_, the dissociation rate constant [2]. According to this model, the ratio k_2_/k_1_ ($\equiv k_{\mathrm{e}}$) corresponds to the reciprocal of affinity, where k_e_ determines potency, and k_2_ determines whether a drug behaves as a full agonist, a partial agonist, or an antagonist (with only vestiges of stimulant action).

**Figure 1:**
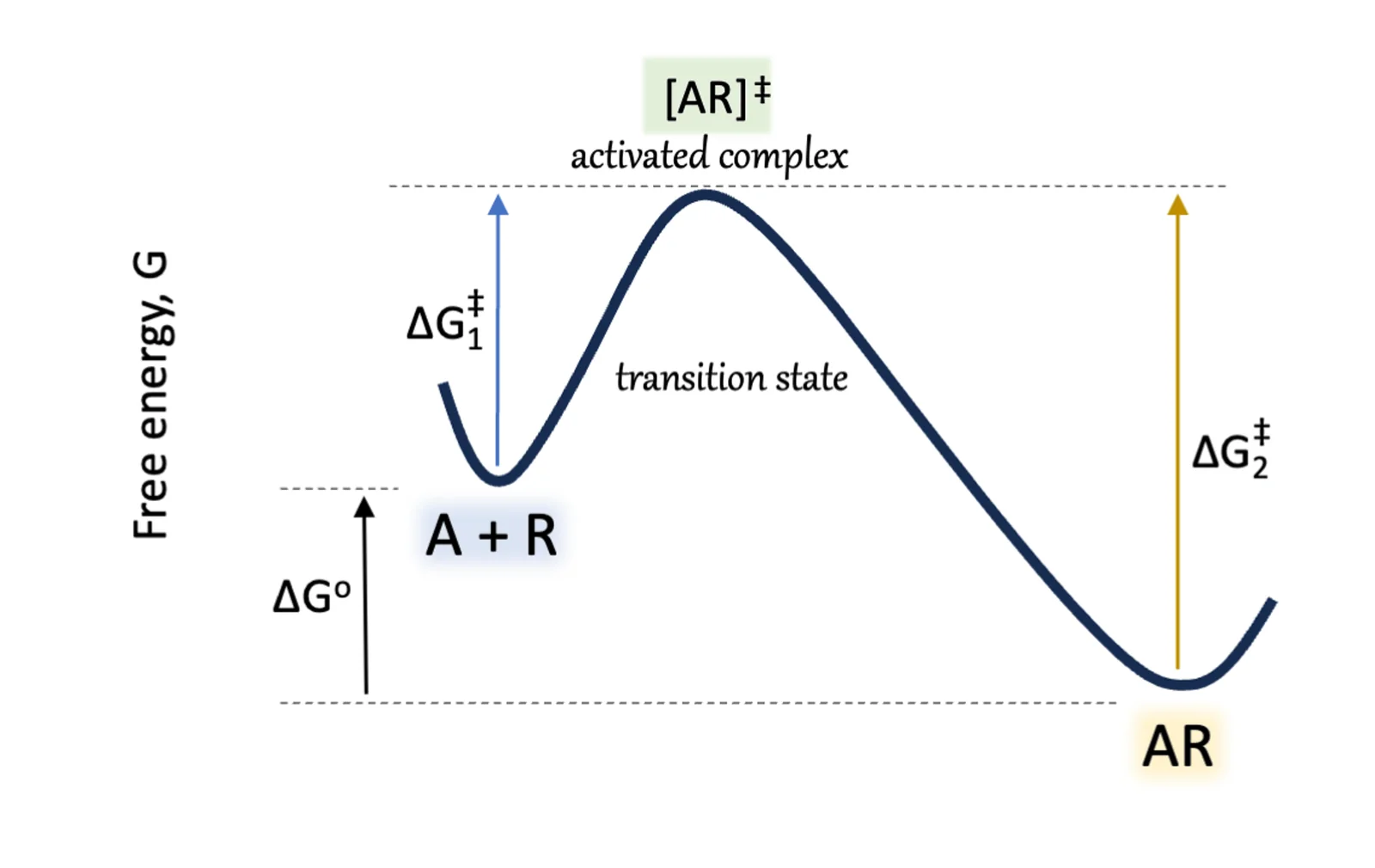
Flux across the association barrier Most drug (A) + receptor (R) encounters do not have enough energy to reach the transition state [AR]^‡^ and simply separate again; only the fraction that crosses goes on to form the drug–receptor complex (AR). Paton's stimulus is the crossing flux, k_1_[A][R], not the amount on either side. Image created by the author using MS PowerPoint (Microsoft Corporation, Redmond, Washington).

Because rate theory is built entirely from k_1_ and k_2_, it is a theory about the ‘height’ and ‘shape’ of the free-energy barriers that separate A + R from AR, not merely about the free-energy difference between (A + R) and AR, or the ‘depth’ of the free-energy ‘well’ that separates them. This fits nicely with transition-state theory in chemistry (the existence of an intermediate ‘activated-complex’ that is a necessary intermediate along the transition from reactants to products) [2]. It is the natural thermodynamic scaffold upon which Paton's kinetic postulate can be built from first principles, as shown herein.

The elementary kinetic schema

Paton's starting point [1,2] was the canonical reversible bimolecular reaction $\mathrm{A} + \mathrm{R} \rightleftharpoons \mathrm{AR} \qquad \bigl[k_1\ (\text{association, } \mathrm{M^{-1}\,s^{-1}}),\ k_2\ (\text{dissociation, } \mathrm{s^{-1}})\bigr]$, with the net rate of drug-receptor complex AR formation being given by $\mathrm{d}[\mathrm{AR}]/\mathrm{d}t = k_1[\mathrm{A}][\mathrm{R}] - k_2[\mathrm{AR}]$. Rather than the cumulative effect of occupation of R by A as in classical theory, rate-theory stimulus, S(t), is proportional to the time-dependent rate of association of A with R, according to $S(t) = \mathrm{const} \times k_{1}[A][R](t)$.

This operational definition is what distinguishes rate theory from classical occupation theory: S(t) depends on an instantaneous rate (units of concentration/time), rather than a dimensionless occupied fraction of receptors. It is important to mention that S(t), as defined here, is Paton's theoretical construct for the receptor-level stimulus, not a directly measured biological or concentration-response variable; the observed tissue response is a downstream, generally nonlinear, transformation of S(t), and should not be equated with it.

Transition-state form of rate theory

Each of the rate constants in rate theory is a rate constant for passage over the free-energy barrier in either the forward or reverse direction (association or dissociation) of the drug-receptor complex AR. The transition-state representations are [15,27]: $k_1 \cong \kappa (k_{\mathrm{B}}T/h) \cdot \exp(-\Delta G^{\ddagger}_{1}/RT) \cong \exp(\Delta S^{\ddagger}_{1}/R) \cdot \exp(-\Delta H^{\ddagger}_{1}/RT)$ and $k_2 \cong \kappa (k_{\mathrm{B}}T/h) \cdot \exp(-\Delta G^{\ddagger}_{2}/RT) \cong \exp(\Delta S^{\ddagger}_{2}/R) \cdot \exp(-\Delta H^{\ddagger}_{2}/RT)$, where ΔG^‡^_1_ is the free energy of activation for the encounter and coupling of $\mathrm{A} + \mathrm{R} \rightarrow [\mathrm{AR}]^{\ddagger}$, ΔG^‡^_2_ is the free energy of activation for the reverse step, uncoupling and separation of $\mathrm{AR} \rightarrow [\mathrm{AR}]^{\ddagger} \rightarrow \mathrm{A} + \mathrm{R}$ (Figure 2). Because k_1_ is a bimolecular (M^-1^s^-1^) rate constant, the prefactor κ(kBT/h), which has units of s^-1^, requires a standard-state concentration term ($C^{\circ} = 1\ \mathrm{M}$ M by convention) to remain dimensionally consistent; the activation free energies reported here are accordingly standard-state free energies referenced to C°.

**Figure 2:**
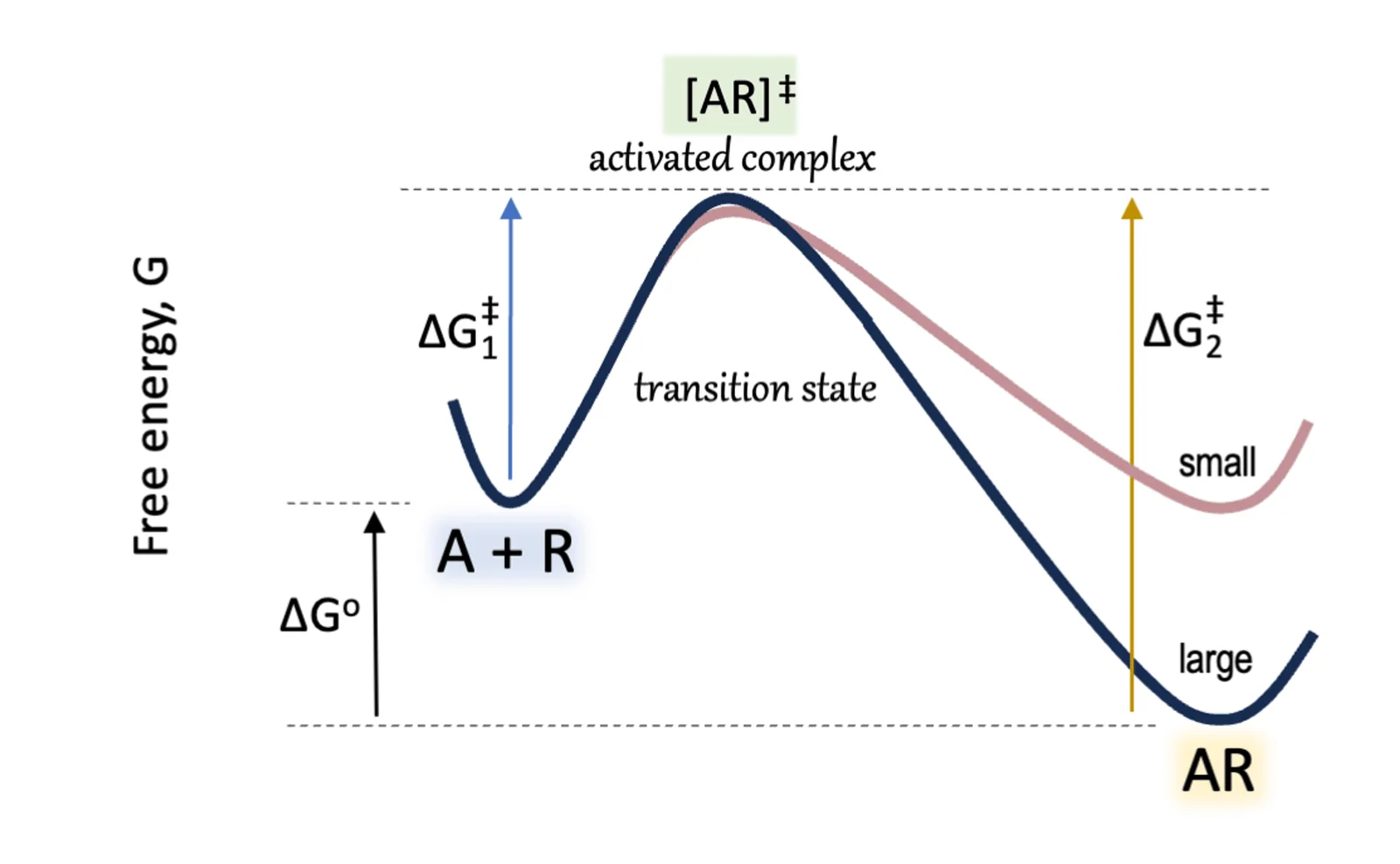
Graphic representation of agonist (shallow well) vs. antagonist (deep well) Free-energy reaction-coordinate profiles for a drug-receptor interaction (Eyring/transition-state representation), comparing a stimulant (shallow well, small ΔG^‡^_2_) with an antagonist-like complex (deep well, large ΔG^‡^_2_). All symbols are defined in the text or Table 1. This is a conceptual model specific to Paton’s rate theory, not a general representation of modern receptor pharmacology, in which efficacy depends on gating as well as binding. Image created by the author using MS PowerPoint (Microsoft Corporation, Redmond, Washington).

Substituting into the stimulus function gives the thermodynamic form of Paton's postulate: $S(t) = \mathrm{const} \times \kappa (k_{\mathrm{B}}T/h) \cdot [\mathrm{A}][\mathrm{R}](t) \cdot \exp(-\Delta G^{\ddagger}_{1}/RT)$. This equation makes explicit what rate theory asserts and what occupation theory does not: that the pharmacologic stimulus is governed by the height of the association barrier, ΔG^‡^_1_, not by the free energy of the bound state. A drug can therefore be a strong stimulant (agonist) by virtue of a low ΔG^‡^_1_ even if its bound complex AR is in a thermodynamically shallow well (weak affinity), and conversely a drug can bind very tightly (deep AR well) yet stimulate poorly if ΔG^‡^_1_ is high (Figure 3). This provides a basis from first principles for what Paton [1,2] postulated is needed as an explanation of a continuum of efficacy partial agonism as part of from agonist to partial-agonist to antagonist.

**Figure 3:**
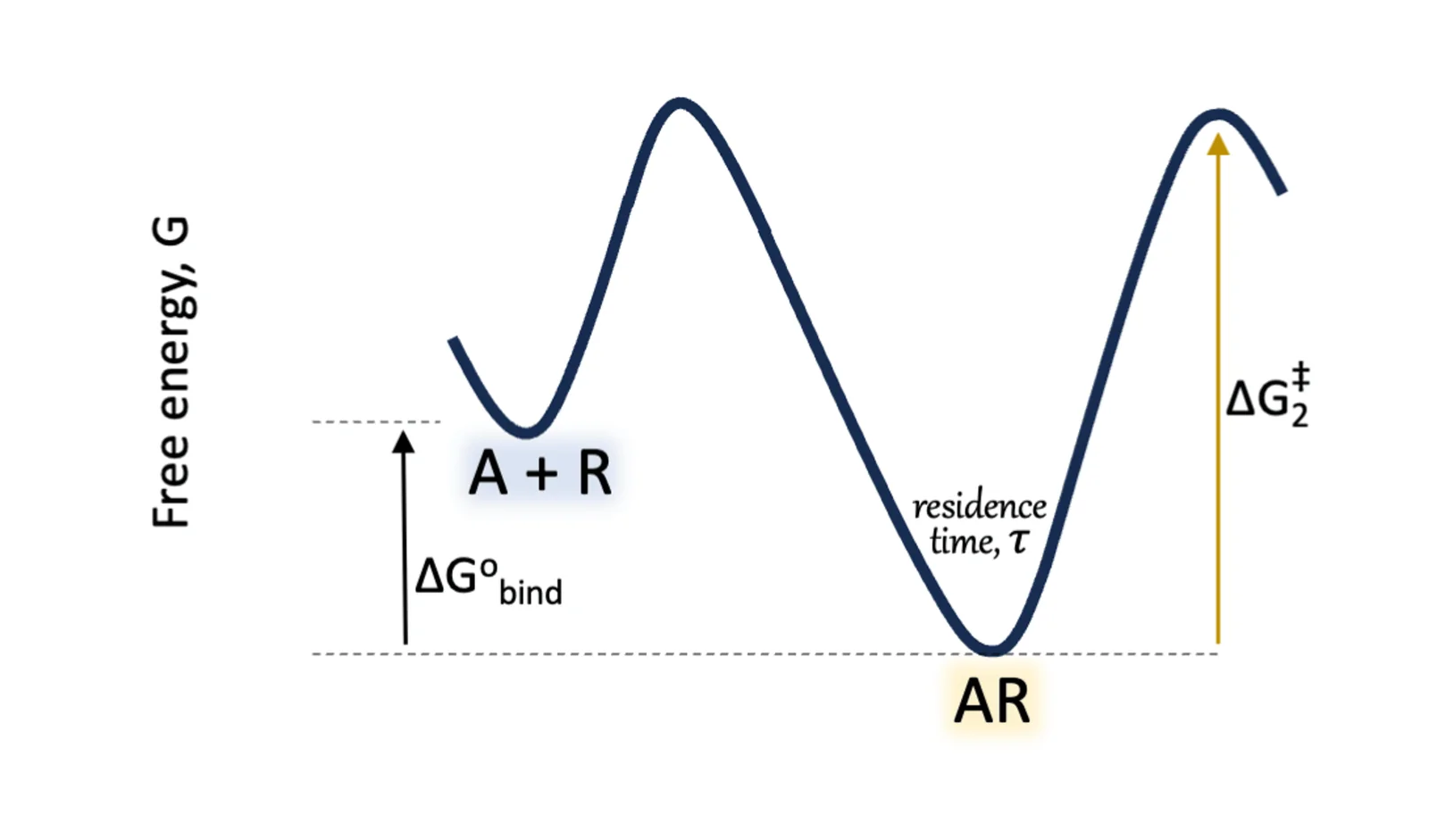
Graphic representation of the AR complex potential well The well depth below the free drug molecule (A) + receptor molecule (R) level is the binding free energy, ΔG°_bind_, which sets K_A_; the separate barrier on the way back out, ΔG^‡^_2_, sets how long the drug stays bound ($\tau = 1/k_2$) — two independent geometric features of the same well. All symbols are defined in the text or Table 1. Image created by the author using MS PowerPoint (Microsoft Corporation, Redmond, Washington).

The free-energy profile and the two rate theory parameters

The overall drug-receptor binding interaction has a standard free energy of binding that is straightforward to write and is well-known: ΔG°_bind_ = RT·ln(K_A_) = -RT·ln(K_A_) = -RT·ln(k_1_/k_2_). Because the forward and reverse activation energies are measured from the same pair of end-states (A + R and AR) relative to the same transition state ([AR]^‡^), they are linked to ΔG°_bind_ by a thermodynamic cycle, readily written $\Delta G^{\circ}_{\mathrm{bind}} = \Delta G^{\ddagger}_{1} - \Delta G^{\ddagger}_{2}$, so that $K_{\mathrm{A}} = k_2/k_1 = \exp[(\Delta G^{\ddagger}_{1} - \Delta G^{\ddagger}_{2})/RT] = \exp(\Delta G^{\circ}_{\mathrm{bind}}/RT)$, which is the same quantity as Paton's reciprocal potency parameter, *k*_e_ ($= k_2/k_1 = \exp(\Delta G^{\circ}_{\mathrm{bind}}/RT)$.

This recovers occupation theory's affinity constant as a derived, equilibrium quantity, while showing that it loses individual information contained in ΔG^‡^_1_ and ΔG^‡^_2_. As a result, two drugs with identical K_A_ (identical ΔG°_bind_) can have completely different absolute values of k_1 _and k_2_, one pair large (fast on, fast off; low barriers on both sides of a shallow energy well) and one pair small (slow on, slow off; high barriers flanking a deep energy well). Occupation theory cannot distinguish these cases at equilibrium. However, rate theory distinguishes them immediately, because S(t) depends on k_1_ (and hence ΔG^‡^_1_) alone, and the pharmacologic character of the drug is set by k_2 _(and hence ΔG^‡^_2_), which governs how fast the stimulus decays once it is formed.

The agonist-antagonist continuum

Writing each activation free energy in enthalpic and entropic parts, $\Delta G^{\ddagger}_{1} = \Delta H^{\ddagger}_{1} - T\Delta S^{\ddagger}_{1}$ and $&Delta;\Delta G^{\ddagger}_{2} = \Delta H^{\ddagger}_{2} - T\Delta S^{\ddagger}_{2}$, allows Paton's qualitative spectrum of drug behavior to be restated as a statement about activation thermodynamics (Table 2).

**Table 2:** Spectrum of efficacy according to rate theory, in terms of free-energy wells In rate theory, a drug's intrinsic activity (efficacy) is not an immutable property but is instead influenced by the receptor with which it forms a complex (AR). Source: the author.

Efficacy	Characteristics	Comments
Full agonist	k_2_ large and ΔG^‡^_2_ small	The AR complex sits in a shallow, easily escaped free-energy well, so that newly formed complexes generate stimulus, then rapidly dissociate, permitting sustained high-frequency signaling
Partial agonist	k_2_ medium and ΔG^‡^_2_ medium	AR complexes dissociate slowly enough to accumulate as unproductive occupancy (functional antagonism of further stimulus), but still contribute some fresh-formation flux
Antagonist	k_2_ small and ΔG^‡^_2_ large	A deep well from which escape is thermodynamically difficult; occupancy persists long after the association flux (and hence the AR stimulus) has decayed to zero, which is exactly Paton's explanation for the persistence of antagonist block and for vestigial agonist action observed transiently upon first application

This spectrum, cast in terms of k_2_ alone, is Paton’s original single-barrier simplification; a complete account of efficacy requires resolving the separate gating step, so that k_2_ (or the corresponding activation free energy) sets residence time and offset kinetics, rather than being the sole biological determinant of a drug’s full pharmacological classification. This is also where rate theory anticipates modern drug-receptor residence-time thinking: $&tau;\ = 1/k_2 = \exp(\Delta G^{\ddagger}_{2}/RT)$ is the mean lifetime of AR, and Copeland and colleagues have shown that in vivo pharmacodynamic duration tracks dissociation kinetics more closely than does the equilibrium constant K_A_ [18,20], a conclusion Copeland later reaffirmed as broadly generalizable across drug-discovery programs [19]. Paton's k_2_ is, in modern language, the reciprocal residence time, and his 1961 [2] argument that a drug's intrinsic activity classification (agonist, partial agonist, antagonist) is determined by k_2_ rather than by K_A_, is a direct conceptual ancestor of the residence-time model [21,22].

Temperature dependence

Because k_1_ and k_2_ are transition-state-theory rate constants, a plot of ln(k/T) against 1/T is linear with slope -ΔH^‡^/R and intercept ln(κk_B_/h) + ΔS^‡^/R (without the quantum constants in this application) [27]. This gives rate theory a testable, quantitative thermodynamics-based prediction that occupation theory (an equilibrium description) does not, namely that the temperature dependence of onset kinetics should separate cleanly into an enthalpic barrier term and an entropic (steric, desolvation, conformational) term, and that these two terms - not K_A_ alone - should track structure-activity relationships in a homologous series of agonists. Modern enthalpy/entropy-resolved binding thermodynamics [14,23] is the direct descendant of this Eyring-plot transition-state logic applied to drug-receptor kinetics.

Occupation theory as the steady-state limit

At equilibrium, $\mathrm{d}[\mathrm{AR}]/\mathrm{d}t = 0$, so $k_1[\mathrm{A}][\mathrm{R}] = k_2[\mathrm{AR}]$, and the forward flux becomes proportional to equilibrium occupancy, according to $S_{\mathrm{eq}} = \mathrm{const} \times k_1[\mathrm{A}] \cdot R_{\mathrm{free}} = k_2[\mathrm{AR}]_{\mathrm{eq}} = k_2 \cdot R_{\mathrm{total}} \cdot p_{\mathrm{A}}$. However, this recovered relationship is not identical to Clark’s original occupation theory, which assumed that response is proportional to occupancy alone, with the same proportionality constant for each drug. Because k_2_ is drug-specific, Seq is proportional to the product k_2_·p_A_ rather than to p_A_ alone, which is Stephenson’s [26] modification of receptor theory, in which each drug has its own efficacy constant multiplying occupancy. Rate theory’s steady-state limit therefore recovers Stephenson’s efficacy-scaled occupancy theory specifically, not Clark’s original, efficacy-free occupancy postulate. Occupation theory is therefore the time-independent limit of rate theory [2] in which the system has been given long enough time to reach steady-state (because of co-occurring association and dissociation, the D-R interaction is at steady-state, rather than true equilibrium). Rate theory is more general than occupation theory, because most physiological responses occur on a timescale comparable to, or shorter than, 1/k_2_, so the system is never actually at the steady state that occupation theory uses. The two theories converge only when k_2_ is fast relative to the observation window - i.e., when the free-energy well of AR is shallow enough that pseudo-equilibrium is reached before the response is measured.

Figure 4 illustrates this transient divergence. Immediately after a step application of agonist, occupancy p_A_(t) rises monotonically from zero toward its equilibrium (steady-state) value, while the stimulus S(t) starts at its maximum (every receptor is still free, so the forward flux is maximal) and falls monotonically toward a smaller steady-state value. For a fast-off agonist-like interaction, the two curves converge quickly; but for a slow-off antagonist-like interaction, some receptors are still occupied, by now impotent molecules, as the stimulus falls off and has all but disappeared, the thermodynamic signature of a drug that is still bound, and still blocking access after it has stopped producing any stimulus after its initial binding.

**Figure 4:**
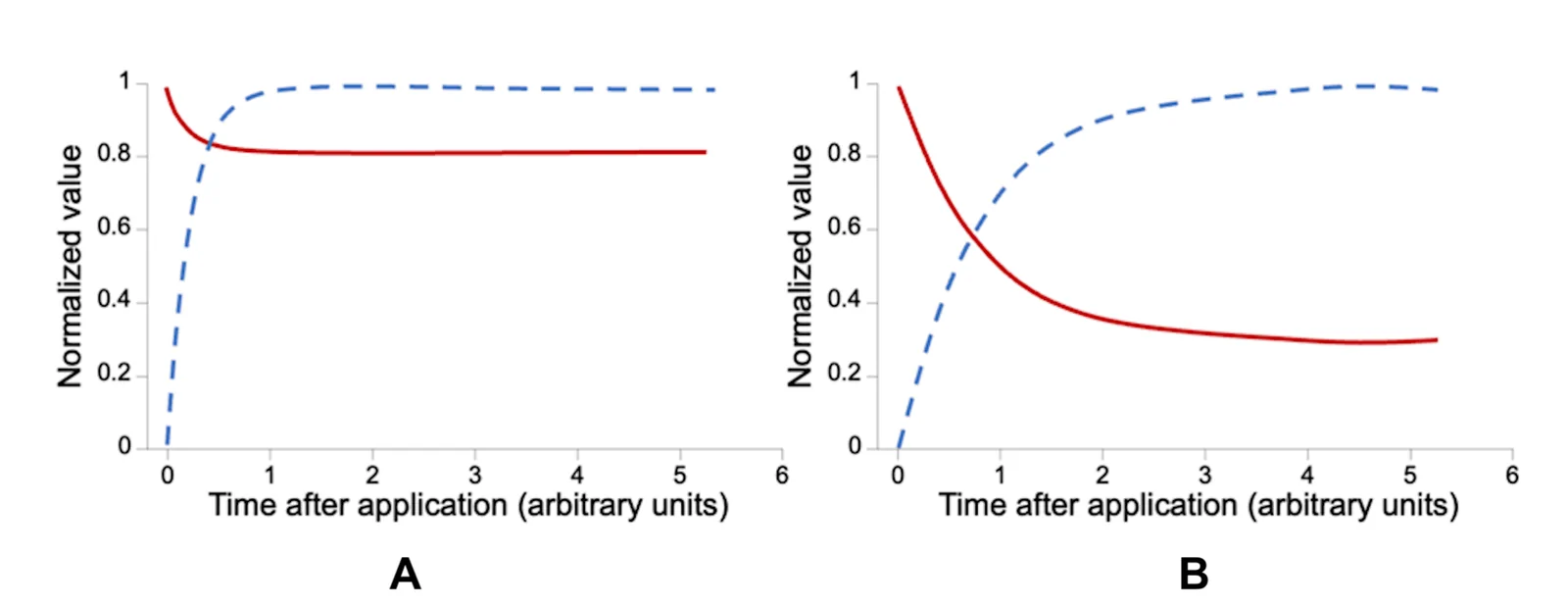
Graphic representation of "agonist-like" and "antagonist-like" behavior Simulated time courses of Paton's stimulus function S(t) (solid line) and equilibrium occupancy p_A_(t) (dashed line) following a step application of an agonist, for a fast-off “stimulant” receptor (A) and a slow-off “antagonist-like” receptor (B). Both curves are normalized to their own asymptotic or initial value for comparison. All symbols are defined in the text or Table 1. Image created by the author using MS PowerPoint (Microsoft Corporation, Redmond, Washington).

Extension I: access-limited kinetics

Paton's original schema [2] treats k_1_ as an intrinsic chemical rate constant for the interaction of A with R, implicitly assuming that A is already available in the receptor's immediate vicinity. In real tissue, the drug must first transit an unstirred diffusion layer, a mucosal or connective-tissue barrier, or the extracellular matrix before it encounters R. Paton and Waud [16] and Paton and Rang [3] showed that this “access” step could be rate-limiting, and that such access-limited kinetics could mimic, or entirely mask, the intrinsic rate constants (k_1_, k_2_) of the drug-receptor interaction (Figure 5).

**Figure 5:**
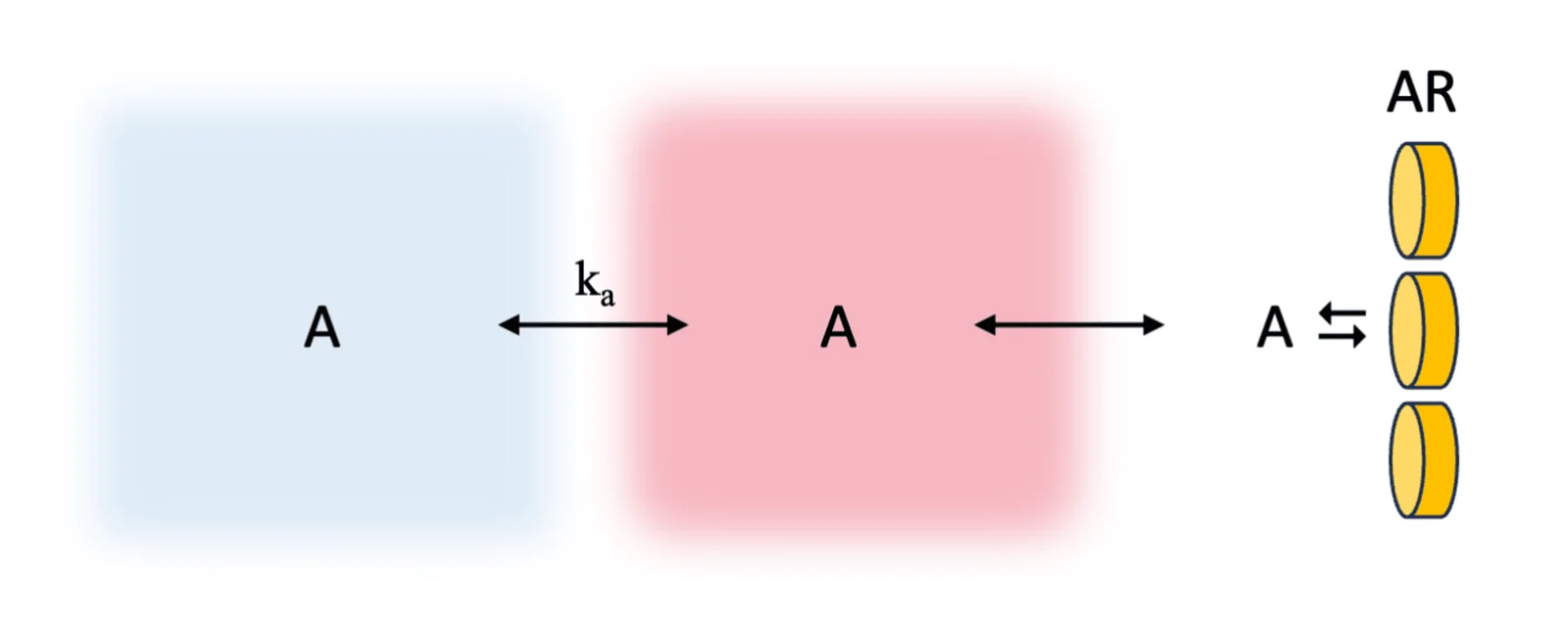
The access step Drug A must first cross a diffusion barrier or unstirred layer into an access compartment near the receptor (rate constant = k_a_, largely entropic) before the intrinsic chemical binding step (k_1_, k_2_) can occur. When k_a_ is rate-limiting, the observed onset of action reflects tissue diffusion geometry rather than receptor chemistry. All symbols are defined in the text or Table 1. Image created by the author using MS PowerPoint (Microsoft Corporation, Redmond, Washington).

Thermodynamically, this is simply represented as a second activated process placed in series with the first.

$\mathrm{A}(\text{bulk solution}) \rightleftharpoons \mathrm{A}(\text{access compartment}) \quad \mathrm{then} \quad \mathrm{A}(\mathrm{access}) + \mathrm{R} \rightleftharpoons \mathrm{AR}$.

Each step, access and receptor binding, is modeled as an independent transition-state rate process with its own ΔG^‡^. Because diffusion through a tissue barrier is overwhelmingly an entropic process (the drug must find a path through a constrained geometry [28]; there is little enthalpic barrier to simple translational diffusion), the access rate constant ka can be approximated by $k_{\mathrm{a}} \approx \exp(\Delta S^{\ddagger}_{\mathrm{access}}/R)$, with $\Delta H^{\ddagger}_{\mathrm{access}} \approx 0$. This is a simplifying assumption, not a general property of diffusion, since access through biological barriers can depend on drug- and tissue-specific factors (e.g., specific membrane transporters, binding to interstitial components, or partition-limited steps), and a measurable enthalpic contribution to access should not be excluded a priori for any given drug-tissue system.

This is the thermodynamic reason that access-limited kinetics are only weakly temperature-dependent compared with a chemical-association step (which usually has a measurable ΔH^‡^_1_). When access is slow relative to the intrinsic binding step, the observed onset rate is no longer fully k_1_[A], but is dominated by k_a_; the tissue-level “potency” and “speed of onset” then become properties of the tissue's diffusion geometry (thickness, lipophilicity, partitioning, unstirred layer, etc.) rather than that of the intrinsic receptor interaction dynamics. Paton and Waud's [16] quantitative analysis of onset and offset kinetics, and Paton's [4] broader kinetic-theory review, are the experimental and theoretical exposition of this two-barrier thermodynamic picture, and the logic remains the conceptual basis of subsequent “micro-pharmacokinetic-pharmacodynamic” analyses of how membrane partitioning shapes the observed k_on_ of a drug at its receptor [28].

Extension II: separating binding from transduction

Paton's original schema treats AR as a single state that either exists or does not, with the stimulus generated directly by the rate of forming the AR complex. Del Castillo and Katz (1957) [17], working from end-plate electrophysiology, showed that this is an oversimplification: binding of agonist to receptor and the conformational change that opens ion channels (or otherwise produces the biological signal) are two chemically distinct, thermodynamically separable steps

$A + R \rightleftharpoons AR \rightleftharpoons AR^{*}$ (binding step: $k_1, k_2$; gating step: $\alpha, \beta$)

where AR* is the active (signaling) conformation. This adds a second free-energy barrier that is associated with the gating or isomerization barrier, which is governed by its own ΔG^‡^_β_ and ΔG^‡^_α_, with an isomerization equilibrium constant $E = \beta/\alpha$ that is conceptually distinct from the binding equilibrium constant $K_{\mathrm{A}} = k_2/k_1$. This decomposition shows where “efficacy” emerges in energetic terms. Whereas K_A_ is determined by the difference of the two binding barriers, efficacy is determined by the difference of the two signal transduction barriers, $\Delta G^{\circ}_{\mathrm{gating}} = \Delta G^{\ddagger}_{\beta} - \Delta G^{\ddagger}_{\alpha}$. A drug can therefore have a very favorable (low) ΔG^‡^_1_ and a deep AR well (high affinity), yet a very unfavorable ΔG^‡^_β_ (poor transduction) - giving it high affinity but low efficacy, which is the thermodynamic explanation of how a substance (ligand) can be a pharmacologic competitive antagonist although chemically similar structurally to an agonist.

Because the act of binding often changes the local protein energetics that determine ΔG^‡^_β_ (e.g., an induced-fit or conformational-selection effect), affinity and efficacy are not in general thermodynamically independent quantities. This is a central argument of Colquhoun [29] and more recently Higham and Colquhoun [30]: binding and transduction share a conformational coordinate, so the free energy measured in a binding assay is influenced by an efficacy-dependent term whenever the ligand is not a pure antagonist. Monod, Wyman, and Changeux's [31] ‘allosteric two-state model’, Black & Leff’s [32] ‘operational model’ of agonism, and De Lean, Stadel, and Lefkowitz's [33] ‘ternary complex model’ for G-protein-coupled receptors are, in thermodynamic terms, different bookkeeping methods for the same underlying physiologic thermodynamic systems: a multi-well free-energy surface with two or more sequential or branching barriers - one for receptor occupancy and one (or more) for the receptor signaling conformation(s). Where a receptor can reach and interact with more than one active conformation, each coupled preferentially to a different intracellular effector, the same molecule can have a different effective ΔG^‡^_gating_ to each pathway, which is the thermodynamic basis of what is now called ‘biased agonism’ or ‘functional selectivity’ [25].

Paton's 1961 [2] theory is thus seen to be the minimal, one-barrier special case of a more general multi-barrier model: it collapses binding and transduction into a single step, because for the rapid, small, positively charged agonists at the neuromuscular junction that motivated it, binding and channel-opening occur on a timescale that was too similar to be resolved by mid-twentieth-century methods. Del Castillo and Katz's [17] two-step schema, and everything built on it since, including receptor desensitization models [34], is the natural refinement obtained once binding and transduction are resolved as separate events with separate free-energy profiles.

## Results

Paton's rate theory can be stated, without loss of content, as the transition-state-theory proposition that: $\mathrm{Stimulus}(t) = \mathrm{const} \times \kappa (k_{\mathrm{B}}T/h) \cdot [\mathrm{A}][\mathrm{R}](t) \cdot \exp(-\Delta G^{\ddagger}_{1}/RT)$, with the drug's pharmacological character (agonist, partial agonist, or antagonist) determined by the reverse barrier, $\text{Offset rate} = \kappa (k_{\mathrm{B}}T/h) \cdot \exp(-\Delta G^{\ddagger}_{2}/RT) = 1/\tau_{\mathrm{residence}}$, and the classical equilibrium affinity recovered as the difference of the two barriers, $K_{\mathrm{A}} = \exp[(\Delta G^{\ddagger}_{1} - \Delta G^{\ddagger}_{2})/RT]$. Extending the theory either in series (e.g., access-limited kinetics) or in sequence (e.g., binding-then-transduction) shows that Paton's original one-barrier schema turns out to be the simplest member of a general family of multi-barrier free-energy surfaces that also includes access-limited pharmacokinetics, the del Castillo-Katz mechanism, the Monod-Wyman-Changeux and ternary complex models, and biased agonism. This reframing shows precisely why rate theory could account for phenomena that a purely equilibrium (occupation) theory could not, such as the excitation-then-block sequence with nicotinic acetylcholine receptor agonists, tachyphylaxis, and the lingering “vestigial” stimulant action of antagonists. Those phenomena are transients governed by ΔG^‡^_1_ and ΔG^‡^_2 _(and, where relevant, ΔG^‡^_β_ and ΔG^‡^_α_) separately, and are invisible to any theory that collapses the drug-receptor interaction into a single equilibrium constant such as K_A_.

## Discussion

The central goal of this manuscript was to show that Paton's rate theory is not a rival empirical alternative to occupation theory, but instead is a special case of a more general thermodynamic description of drug-receptor interactions, in which occupation theory is seen to be the steady-state limit of rate theory.

The underlying model of classical and rate theories is well-known mass-action kinetics. For any two-state model such as $K_{\mathrm{A}} = \exp[(\Delta G^{\ddagger}_{1} - \Delta G^{\ddagger}_{2})/RT]$, setting $\mathrm{d}[\mathrm{AR}]/\mathrm{d}t = 0$ automatically returns the standard occupancy expression. Paton made this point essentially as a validation of rate theory. He measured onset/offset rates of antagonism kinetically to get k₁ and k₂, and reported that "values of k₂/k₁ so obtained agreed with those obtained from the intensity of antagonism at equilibrium," i.e., he explicitly checked that the kinetic ke reduces to, and numerically matches, the classical equilibrium affinity constant. Later reviews of receptor theory [35] point out that rate theory is a generalization that recovers classical theory at equilibrium. So the mathematical kinetics-based derivations used herein are not new; however, the Eyring transition-state/ΔG^‡^ approach [15], as well as the representation of k₁ and k₂ as transition-state rate constants and defining K_A_ in those terms, $K_{\mathrm{A}} = \exp[(\Delta G^{\ddagger}_{1} - \Delta G^{\ddagger}_{2})/RT]$, has not been posited before to the author’s knowledge. = exp[(ΔG^‡^1 − ΔG^‡^2)/RT] has not, to the author’s knowledge, been posited in exactly this algebraic form before. Transition-state, free-energy-barrier framing of drug-receptor k_on_ and k_off_ individually, however, is well established in the modern computational residence-time literature [36-38], and the present derivation merely makes that connection explicit for Paton’s original two-parameter kinetic schema, rather than introducing transition-state reasoning to residence-time pharmacology for the first time.

Limitations and future directions

The present framework has limitations. The core derivation treats k_1_ and k_2_ as elementary, single-barrier transition-state rate constants; in reality, many drug-receptor interactions proceed through a diffusional encounter complex, possibly multiple conformational substates, and solvent-reorganization steps that are lumped into the apparent k_1_ and k_2_ measured experimentally. The extensions partially address this by explicitly resolving one additional step at a time, but a fully resolved multi-step free-energy surface, of the kind obtained from molecular-dynamics free-energy calculations [5] or single-molecule kinetics [39], would in general require more than the two or three barriers considered here. The transmission coefficient κ was taken as 1, as is common, throughout; recrossing effects, non-adiabatic transitions, and tunneling were not considered, though these are unlikely to dominate non-quantum aspects of drug-receptor interactions. This means that the apparent ΔG^‡^_1_ and ΔG^‡^_2_ extracted from experimental k_1_ and k_2_ are composite barriers averaged over these hidden steps, so the enthalpy-entropy decomposition characterizes the rate-limiting apparent barrier rather than a single elementary chemical event; rebinding, in particular, can inflate the apparent residence time beyond 1/k_2_ for the intrinsic dissociation step alone [21,22].

Future work could modify/advance upon the current choices and approach. The schematic, non-quantitative free-energy reaction-coordinate diagram was constructed by piecewise cosine interpolation between illustrative end-point and transition-state energies; it is intended to convey the qualitative relationship between well depth, barrier height, and pharmacological category, not to represent any measured energy values. The simulated divergence figure was generated by analytically solving the linear ordinary differential equation $\mathrm{d}[\mathrm{AR}]/\mathrm{d}t = k_1[\mathrm{A}](R_{\mathrm{total}} - [\mathrm{AR}]) - k_2[\mathrm{AR}]$ for a step change in [A] at *t = 0*, giving $[\mathrm{AR}](t) = [\mathrm{AR}]_{\mathrm{eq}} \cdot (1 - \exp(-k_{\mathrm{obs}} \cdot t))$ with $k_{\mathrm{obs}} = k_1[\mathrm{A}] + k_2$. Stimulus was computed as $S(t) = k_1[\mathrm{A}] \cdot (R_{\mathrm{total}} - [\mathrm{AR}](t))$ and occupancy as $p_{\mathrm{A}}(t) = [\mathrm{AR}](t)/R_{\mathrm{total}}$. Likewise, two parameter regimes were chosen purely for illustration ($k_1[\mathrm{A}] = 1$ in arbitrary rate units; $k_2 = 3$ for the “stimulant” (agonist) case and $k_2 = 0.05$ for the “antagonist-like” case) to display the qualitative divergence between the two functions rather than to fit a specific experimental system.

## Conclusions

Rate theory and classical occupation theory have long been presented as competing accounts of drug action. However, as demonstrated here, viewed under overarching transition-state theory, they are not competitors at all: occupation theory is the equilibrium limit of rate theory, and the parameter that principally distinguishes an agonist from a partial agonist from an antagonist, rate theory's k_2_, is simply the reciprocal of the drug-receptor residence time. Extending the same free-energy logic in series (in order to incorporate ligand diffusion to the receptor) and in sequence (in order to separate binding from transduction) reveals - as special cases of a single thermodynamic framework - the del Castillo-Katz mechanism, the MWC (Monod-Wyman-Changeux) and ternary complex models, and biased agonism. In summary, the basis of rate-based theories of the drug-receptor interaction - that a drug's action is not fully specified by how tightly it binds but by the separate heights of the free-energy barriers that govern how it associates with, and dissociates from, its receptor - is seen to have a first-principles foundation.

## References

[1] Paton WDM (1960). The principles of drug action. Proc R Soc Med.

[2] Paton WDM (1961). A theory of drug action based on the rate of drug-receptor combination. Proc Royal Soc London Series B.

[3] Paton WDM, Rang HP (1965). The uptake of atropine and related drugs by intestinal smooth muscle of the guinea-pig in relation to acetylcholine receptors. Proc R Soc Lond B Biol Sci.

[4] Paton WDM (1967). Kinetic theories of drug action with special reference to the acetylcholine group of agonists and antagonists. Annals of the New York Acad Sci.

[5] Ariëns EJ (1954). Affinity and intrinsic activity in the theory of competitive inhibition. I. Problems and theory. Arch Int Pharmacodyn Ther.

[6] Clark AJ (1926). The reaction between acetyl choline and muscle cells. J Physiol.

[7] Gaddum JH (1957). Theories of drug antagonism. Pharmacol Rev.

[8] Gero A (1977). Use of the biological stimulus in determining parameters of drug action, and its relationship to the drug effect: a contribution to the theory of drug action. J Theor Biol.

[9] Gero A (1978). Biological stimulus and drug effect. J Theor Biol.

[10] Hill AV (1909). The mode of action of nicotine and curari, determined by the form of the contraction curve and the method of temperature coefficients. J Physiol.

[11] Maehle AH (2004). "Receptive substances": John Newport Langley (1852-1925) and his path to a receptor theory of drug action. Med Hist.

[12] Maehle AH, Prüll CR, Halliwell RF (2002). The emergence of the drug receptor theory. Nat Rev Drug Discov.

[13] Nickerson M (1956). Receptor occupancy and tissue response. Nature.

[14] Raffa RB (2001). Drug-Receptor Thermodynamics: Introduction and Applications. https://cir.nii.ac.jp/crid/1971149384867778949.

[15] Eyring H (1935). The activated complex in chemical reactions. J Chem Phys.

[16] Paton WD, Waud DR (1964). A quantitative investigation of the relationship between rate of access of a drug to receptor and the rate of onset or offset of action. Naunyn Schmiedebergs Arch Exp Pathol Pharmakol.

[17] Del Castillo J, Katz B (1957). Interaction at end-plate receptors between different choline derivatives. Proc R Soc Lond B Biol Sci.

[18] Copeland RA, Pompliano DL, Meek TD (2006). Drug-target residence time and its implications for lead optimization. Nat Rev Drug Discov.

[19] Copeland RA (2016). The drug-target residence time model: a 10-year retrospective. Nat Rev Drug Discov.

[20] Tummino PJ, Copeland RA (2008). Residence time of receptor-ligand complexes and its effect on biological function. Biochemistry.

[21] Vauquelin G (2010). Rebinding: or why drugs may act longer in vivo than expected from their in vitro target residence time. Expert Opin Drug Discov.

[22] Vauquelin G, Charlton SJ (2010). Long-lasting target binding and rebinding as mechanisms to prolong in vivo drug action. Br J Pharmacol.

[23] Freire E (2008). Do enthalpy and entropy distinguish first in class from best in class?. Drug Discov Today.

[24] Kenakin T (2026). Biased signaling as allosteric probe dependence: everything old is new again. Biochemistry.

[25] Kenakin T, Christopoulos A (2013). Signalling bias in new drug discovery: detection, quantification and therapeutic impact. Nat Rev Drug Discov.

[26] Stephenson RP (1956). A modification of receptor theory. Br J Pharmacol Chemother.

[27] Zhou HX (2010). Rate theories for biologists. Q Rev Biophys.

[28] Zhu Q, Lu Y, He X (2017). Entropy and polarity control the partition and transportation of drug-like molecules in biological membrane. Sci Rep.

[29] Sykes DA, Dowling MR, Leighton-Davies J (2012). The influence of receptor kinetics on the onset and duration of action and the therapeutic index of NVA237 and tiotropium. J Pharmacol Exp Ther.

[30] Colquhoun D (1998). Binding, gating, affinity and efficacy: the interpretation of structure-activity relationships for agonists and of the effects of mutating receptors. Br J Pharmacol.

[31] Higham JP, Colquhoun D (2024). The affinity-efficacy problem: an essential part of pharmacology education. R Soc Open Sci.

[32] Monod J, Wyman J, Changeux JP (1965). On the nature of allosteric transitions: a plausible model. J Mol Biol.

[33] Black JW, Leff P (1983). Operational models of pharmacological agonism. Proc R Soc Lond B Biol Sci.

[34] De Lean A, Stadel JM, Lefkowitz RJ (1980). A ternary complex model explains the agonist-specific binding properties of the adenylate cyclase-coupled beta-adrenergic receptor. J Biol Chem.

[35] Katz B, Thesleff S (1957). The interaction between edrophonium (tensilon) and acetylcholine at the motor end-plate. Br J Pharmacol Chemother.

[36] Waud DR (1968). Pharmacological receptors. Pharmacol Rev.

[37] Dror RO, Pan AC, Arlow DH (2011). Pathway and mechanism of drug binding to G-protein-coupled receptors. Proc Natl Acad Sci U S A.

[38] Schuetz DA, de Witte WE, Wong YC (2017). Kinetics for drug discovery: an industry-driven effort to target drug residence time. Drug Discov Today.

[39] van Oijen AM (2011). Single-molecule approaches to characterizing kinetics of biomolecular interactions. Curr Opin Biotechnol.

